# Predictive Modeling of Covid-19 Data in the US: Adaptive Phase-Space Approach

**DOI:** 10.1109/OJEMB.2020.3008313

**Published:** 2020-07-09

**Authors:** Vasilis Z. Marmarelis

**Affiliations:** Department of Biomedical EngineeringUniversity of Southern California 5116 Los Angeles CA 90089 USA

**Keywords:** Adaptive modeling of Covid-19 time-series data, epidemiological predictive modeling, riccati-based phase-space modeling, statistical detection of Covid-19 infection waves

## Abstract

There are currently intensified efforts by the scientific community world-wide to analyze the dynamics of the Covid-19 pandemic in order to predict key epidemiological effects and assist the proper planning for its clinical management, as well as guide sociopolitical decision-making regarding proper mitigation measures. Most efforts follow variants of the established SIR methodological framework that divides a population into “Susceptible”, “Infectious” and “Recovered/Removed” fractions and defines their dynamic inter-relationships with first-order differential equations. *Goal:* This paper proposes a novel approach based on data-guided detection and concatenation of infection waves – each of them described by a Riccati equation with adaptively estimated parameters. *Methods:* This approach was applied to Covid-19 daily time-series data of US confirmed cases, resulting in the decomposition of the epidemic time-course into five “Riccati modules” representing major infection waves to date (June 18th). *Results:* Four waves have passed the time-point of peak infection rate, with the fifth expected to peak on July 20th. The obtained parameter estimates indicate gradual reduction of infectivity rate, although the latest wave is expected to be the largest. *Conclusions:* This analysis suggests that, if no new waves of infection emerge, the Covid-19 epidemic will be controlled in the US (<5000 new daily cases) by September 26th, and the maximum of confirmed cases will reach 4,160,000. Importantly, this approach can be used to detect (via rigorous statistical methods) the emergence of possible new waves of infections in the future. Analysis of data from individual states or countries may quantify the distinct effects of different mitigation measures.

## I. Introduction

Many efforts have been made recently to analyze the time-course of the Covid-19 pandemic daily data in various countries or regions and to predict key aspects of its eventual growth in order to assist the proper planning for healthcare resources and related socioeconomic decision-making. Among them, dominant role is played by the SIR class of compartmental epidemiological models, introduced about a century ago by Kermack and McKendrick [1], and its many variants over the years [2]–[5] that generally utilize compartments of “**S**usceptible”, “**I**nfectious” and “**R**emoved” fractions of the population, which are interconnected with dynamic relationships described by nonlinear ordinary differential equations. Another commonly used approach employs linear Auto-Regressive Integrated Moving-Average (ARIMA) models that have been popular in econometrics [6]. From the policy-planning point of view, practical importance is attained by *predictive* modeling methods that can provide reliable estimates of key parameters of the unfolding infectious process at each point in time on an adaptive basis, as well as offer useful insights into the dynamic structure of the infectious process. For example, such adaptive methods can offer useful predictions of the maximum number of total infections and an upper bound of the daily confirmed new cases for the purpose of planning the proper clinical management of the epidemic. Furthermore, the obtained model should be interpretable in terms of the dynamic characteristics of the epidemic process (e.g. infectivity rate etc.) in order to assist policy planning and operational implementation. From these observations arise two key aspects of a desirable modeling approach:

1) *Suitable model form:* The employed model form must capture the essential dynamic characteristics of the epidemic process at each time-point in a manner that is scientifically interpretable and operationally useful.
2) *Robust estimation and adaptive modeling:* Robust estimation of the model parameters at each time-point must be feasible using tested statistical methods in a manner that can detect possible changes in the underlying modeling assumptions over time and offer the means for model adaptation.

If these two key aspects can be secured, then it would be possible to predict the maximum spread of anticipated infections and the maximum rate of infections (as well as their respective timing) in order to assist rational decision-making.

This paper presents one such approach that employs an adaptive modeling/estimation strategy based on the use of *concatenated Riccati-type modules* (each described by a parabolic phase-space representation) and suitable adaptive statistical estimation methods. The potential utility of this approach is initially demonstrated with the adaptive analysis of daily data of reported Covid-19 confirmed cases in the US up to the present time (June 18, 2020).

The extensive literature on the subject of epidemiological modeling is not reviewed here in the interest of space, but some basic comparisons of the proposed approach with the most widely-used SIR class of models will be discussed. Some representative recent modeling applications to Covid-19 data that may be of interest to the readers include: a simulation study of the SEIR model (a variant of the SIR model that includes a compartment for “Exposed” individuals) for Covid-19 in Northern Italy [7], a model that seeks to estimate the transmission risk of the epidemic [8], and a model for the spread of the epidemic in China [9]. There are many Covid-related modeling studies that have been posted as “preprints under review”, thus more citations will soon be available.

## II. Materials and Methods

The key modeling element of the proposed approach is the “Riccati module” (RM) that is defined by the Riccati Equation (1) with constant coefficients ***A*** and ***B*** (defining a quadratic relation between the rate of change and the number of infections ***X(t)*** at each time) [10]. The additive stochastic term ***R(t)*** represents all unknown random influences (unknown external factors and errors/noise) affecting the reported time-series data [11]–[15]:

\begin{equation*}
\boldsymbol{dX}(\boldsymbol{t})/\boldsymbol{dt} = \boldsymbol{AX}(\boldsymbol{t})-\boldsymbol{B}{\boldsymbol{X}^{{\bf 2}}}(\boldsymbol{t}) + \boldsymbol{R}(\boldsymbol{t})\tag{1}
\end{equation*}

This equation captures the essential *self-limiting* aspect of an infectious process (due to the gradually acquired “herd immunity” and countervailing factors) in a relatively simple manner by considering the “effective rate” (which relates the derivative to the function) being reduced linearly with rising ***X(t)*** as: ***[A – BX(t)],*** instead of being a constant as in the conventional rate processes of the form: ***dX(t)/dt = AX(t)***.

Thus, the parameter ***A*** is the initial “infectivity rate constant” that is dominant in the initial exponential-like growth of the infection and quantifies the degree of contagiousness of an infectious agent along with the level of contagious interactions in a given “infection pool” (IP). On the other hand, the parameter ***B*** depends on the size of susceptible population in the IP and also quantifies the degree to which the aforementioned acquired “herd immunity” and countervailing factors (both natural and socially or administratively imposed by the infected community) constrain the initial rapid growth of the infection and eventually achieve its control. This process is described by a sigmoidal curve defined by Equation (2), which is the general solution of the Riccati Equation (1) (in the absence of random perturbations ***R(t)***), where the maximum number ${\boldsymbol X_{\max}}$ of total infections anticipated by the Riccati model (i.e. the plateau of the sigmoidal curve) is given by the ratio of the two parameters ${\boldsymbol X_{\max}= (A/ B)}$:

\begin{equation*}
\boldsymbol {X} \left(\boldsymbol {t} \right) = {\boldsymbol {X}_{\boldsymbol {\max}}}/ \left[ {1 + \boldsymbol{K}\ \mathbf {exp} \left( - \boldsymbol {At} \right)} \right]\tag{2}
\end{equation*}

where ${\boldsymbol K= [ (X_{\max}/ X_{\rm in}) -1] }$, with ${\boldsymbol X_{\rm in}}$ being the initial value of ***X(t)*** at the start of the respective RM single-pool infection. The two parameters, ***A*** and ***B***, of each RM attain useful interpretations that offer insights into the dynamic characteristics of the infectious process, which is generally decomposed into a cascade of RMs estimated via the proposed adaptive methodology and representing the ongoing “recruitment” of distinct/major IPs. This model-derived knowledge may assist the effective management of an epidemic describable by a model composed of such concatenated (latent) RMs.

It is clearly desirable to obtain reliable “running” estimates of these parameters from time-series data (e.g. daily Covid-19 data) at any given point in time. The Riccati-equation model has been shown previously to represent self-limiting infectious processes that are confined within *single isolated* “infection pools” (IPs) [11]–[15]. The challenge in the study of the Covid-19 epidemic is that, due to its highly contagious nature, there are multiple communicating IPs that are recruited during the course of the epidemic and contribute to the reported data at the respective national, international or multi-community level. This presents us with the daunting task of separating the superimposed sigmoidal time-courses of multiple RMs corresponding to the various IPs (without the benefit of separate data from individual IPs). To perform this task, we propose a methodology that utilizes an adaptive estimation procedure to detect (via a “running” statistical Hypothesis test) and separate the concatenated parabolic phase-space representations of the RMs that are present in the data up to a given time-point.

The phase-space representation of a dynamic process ***X(t)*** pertains to the relation between ***X(t)*** and its derivative over time (in the absence of random perturbations). The Riccati Equation (1) indicates that this relation is parabolic. For discrete-time data (i.e. Covid-19 confirmed cases) up to time-step ***n***, a cascade of parabolic phase-plots can be fitted to the available phase-space data, and estimates of all the parameters ***A*** and ***B*** at each time-step ***n*** can be obtained. These parameter estimates can be used to predict the multi-sigmoidal time-course of the infectious process according to a superposition of cascaded sigmoidal curves, each described by Equation (2) with its distinct parameters. This estimation task begins with the statistical detection and estimation of the first RM that is described by the discretized Riccati-model:

\begin{equation*}
\boldsymbol{\Delta X}\left(\boldsymbol{n} \right)/\boldsymbol{\Delta T} = \boldsymbol{AX}\left(\boldsymbol{n} \right)-\boldsymbol{B}{\boldsymbol{X}^{{\bf 2}}}\left(\boldsymbol{n} \right) + \boldsymbol{R}\left(\boldsymbol{n} \right)\tag{3}
\end{equation*}

where: ***ΔX(n) = [X(n) − X(n* − 1*)]***, and ***ΔT*** denotes the fixed time-step (1 day in this case). Following adaptive estimation of the first RM (see below), we perform statistical Hypothesis testing (using a properly constructed F-statistic) at each time-step to detect the possible emergence of another RM and, if such is detected, then estimate the distinct parameters of the two RMs and separate the contributions to the total reported cases (see below). This procedure is repeated at each time-step ***n*** until all daily data have been analyzed to obtain adaptive estimates of the distinct RM parameters ***A*** and ***B*** that correspond to all detected RMs.

Regarding the *robust* estimation of the parameters ***A*** and ***B***, initial analysis indicated that the standard deviation of the residual values ***R(n)*** depends roughly linearly on ***X(n)***. This non-stationary residual variance implies that least-*squares* fitting of the model of Equation (3) would yield unreliable parameter estimates***.*** However, reliable estimates of ***A*** and ***B*** may be obtained via least-squares regression of the “Normalized Rate of Change”: ***ΔX(n)/X(n)***, (equivalent to the logarithmic derivative) upon ***X(n)*** according to the equation:

\begin{equation*}
\boldsymbol{\Delta X}\left(\boldsymbol{n} \right)/\boldsymbol{X}\left(\boldsymbol{n} \right) = \boldsymbol{A}-\boldsymbol{BX}\left(\boldsymbol{n} \right) + \boldsymbol{R}\left(\boldsymbol{n} \right)/\boldsymbol{X}\left(\boldsymbol{n} \right)\tag{4}
\end{equation*}

when ***ΔT*** in Equation (4) is set to 1 (one day). Since the residual term: ***R(n)/X(n)***, is expected to have (approximately) stationary standard deviation, reliable parameter estimates can be obtained at each time-step ***n***. Furthermore, the “slope parameter” ***B*** in Equation (4) can be evaluated for statistical significance at each time-step ***n*** (by testing the Null Hypothesis that the slope parameter is not significantly different from zero at a specified confidence level) to assess whether Equation (4) remains an appropriate representation of the data. When this Null Hypothesis gets rejected at some time-step ***n***, then adaptive parameter estimates can begin to be used for adaptive prediction of the sigmoidal course of the infection accounted by the respective RM.

This adaptive estimation procedure can be repeated at each time-point ***n***, until the linear relationship expressed by Equation (4) ceases to represent the time-evolution of the data – an event identified adaptively by examining the statistical significance of the reduction in Residual Variance (using Hypothesis testing with an F-statistic) of the regression of the “Normalized Rate of Change” values: [***ΔX(n)/X(n)***] upon the linear relationship of Equation (4) *versus* a second-degree polynomial expression that would indicate the emergence of a new RM. Note that a second-degree polynomial expression like the one in Equation (5) (starting with a positive value at X = 0, since ***A*** must be positive) may not have a zero-crossing in the phase-plot of the “Normalized Rate of Change”, but this is not necessary because it simply quantifies the divergence from the Null Hypothesis (as an Alternative Hypothesis) and does not seek to represent the dynamic characterisitcs of the infectious process. Thus, we construct an adaptive statistical test using the Alternative Hypothesis that the Normalized Rate of Change follows the quadratic model of Equation (5):

\begin{align*}
\boldsymbol{\Delta X}\left(\boldsymbol{n} \right)/\boldsymbol{X}\left(\boldsymbol{n} \right) = &\boldsymbol{A}-\boldsymbol{BX}\left(\boldsymbol{n} \right)\\
&-\boldsymbol{C}{\boldsymbol{X}^2}\left(\boldsymbol{n} \right) + \boldsymbol{R}\left(\boldsymbol{n} \right)/\boldsymbol{X}\left(\boldsymbol{n} \right)\tag{5}
\end{align*}

to be tested at each time-point against the Null Hypothesis of the linear model of Equation (4). For this statistical Hypothesis testing, we use the following F-statistic (with 1 and (N-3) degrees of freedom) that represents the normalized reduction in Residual Variance between the linear and the quadratic expressions (4) and (5):

\begin{equation*}
{\boldsymbol{F}_{{{\bf 1}},\boldsymbol{N} - {{\bf 3}}}} = \left({\boldsymbol{N} - 3} \right){\boldsymbol{Q}_1}/({\boldsymbol{Q}_2}\tag{6}
\end{equation*}

where ***Q*_1_**and ***Q*_2_**denote the computed Residual Variances for the linear and the quadratic expression, respectively, and ***N*** is the number of data-points used in the regression.

The computed ${\boldsymbol F_{1,N-3}}$ is compared at each time-point to the proper critical value ${\boldsymbol F_{crit}}$ (for a significance level of 95%). When ${\boldsymbol F_{1, N-3}> F_{crit}}$, the Null Hypothesis is rejected (at 95% confidence level) and a new RM is deemed to be emerging and included in the model by separating its contributions (and parameters) from those of all other previous RMs. The contributions of all concatenated RM model components are included in the total model prediction. The application of this approach is demonstrated in the following section using daily reported data of Covid-19 confirmed cases in the US from March 11 until June 18 (the completion date of this manuscript), while the epidemic is still ongoing.

## III. Results

We analyzed the publicly reported data of daily new Covid-19 confirmed cases in the US (database curated by Johns Hopkins University) and the cumulative number of confirmed cases since March 11th 2020 (the day the cumulative cases first exceeded 1000 in the US) until June 18th 2020 (the completion date of this manuscript), a period that covers a total of 100 days. Application of the aforementioned methodology identified *five* latent Riccati modules (RM) with distinct parameters ***A*** and ***B*** that are given in Table I, along with the parameters ***K*** of Equation (2) and the respective predictions of the maximum number of anticipated cumulative cases due to each RM model component. Some other key parameters of the five component RMs (e.g. the size and timing of the peak infection rate for each RM) are also reported in Table I. The timing of the peak infection rate (***PIR***) for each RM is given by the expression:

\begin{equation*}
{\boldsymbol{T}_{\boldsymbol{PIR}}} = \boldsymbol{ln}\left(\boldsymbol{K} \right)/\boldsymbol{A}\tag{7}
\end{equation*}

and the corresponding ***PIR*** is determined by A and B as:

\begin{equation*}
\boldsymbol{PIR} = {\boldsymbol{A}^{{\bf 2}}}/\left({{{\bf 4}}\boldsymbol{B}} \right)\tag{8}
\end{equation*}

**TABLE I:** Estimated Parameters of the RM Model Components

***Parameter***	**RM #1**	**RM #2**	**RM #3**	**RM #4**	**RM #5**
***A***	0.39	0.25	0.12	0.08	0.05
***B*** *x 10^−6^*	5.6	0.65	0.14	0.13	0.02
***K***	480	710	280	530	760
${\boldsymbol X_{\max}\,}\,x\,10^{3}$	70	380	820	610	2,280
***PIR*** *x 10^3^*	6.8	23.7	24.6	12.2	28.5
${\boldsymbol T_{PIR}}$	16	26	47	78	132
${\boldsymbol T_{det}}$	8	15	23	37	60

Equation (8) indicates the strong dependence of ***PIR*** on ***A***. Since the ***PIR*** value is critical for planning the clinical management of the pandemic (lest the finite resources of the healthcare system be temporarily overwhelmed). Equation (8) underlines the importance of minimizing (i.e. controlling) ***A*** for a given IP size ${\boldsymbol X_{\max}= A/ B}$. All these parameter estimates are given in Table I for the five RMs, along with the time of their earliest detection ${\boldsymbol T_{det}}$ by the proposed algorithm. The units of these parameter values are the following: ***A*** (days^−1^), ***B*** (days^−1^ cases^−1^), ***K*** (unitless), ${\boldsymbol X_{\max}}$ (cases), ***PIR*** (cases/day), ${\boldsymbol T_{PIR}}$ and ${\boldsymbol T_{det}}$ (days since March 11th 2020).

 The declining values of the estimated parameters ***A*** for the five RMs indicate that *there is gradual reduction of the infectivity rate*, which may be partially due to the effect of the imposed social-distancing and other mitigation measures (see Discussion). These parameter values are updated on a daily basis but were shown to be rather stable away from the days of introduction of new RMs. The estimated parameters ***B*** for the five RMs depend inversely on the size of the susceptible and exposed population in the respective “infection pool” in combination with the effect of mitigation measures (see Discussion). This is consistent with the model-predicted maximum numbers of confirmed cases for the five RMs. The total maximum number of cumulative confirmed cases that is predicted by these five RM components of the model is: 4,160,000 (substantially higher than the current cumulative total of 2,191,100 cases). Of course, this prediction is contingent upon the assumption that no new infection waves will occur and be detected by the algorithm in the future. In connection with this assumption, we note that the F-statistic is rising recently and is approaching the critical value that may trigger the detection of a new emergent infection wave.

**Fig. 1.**
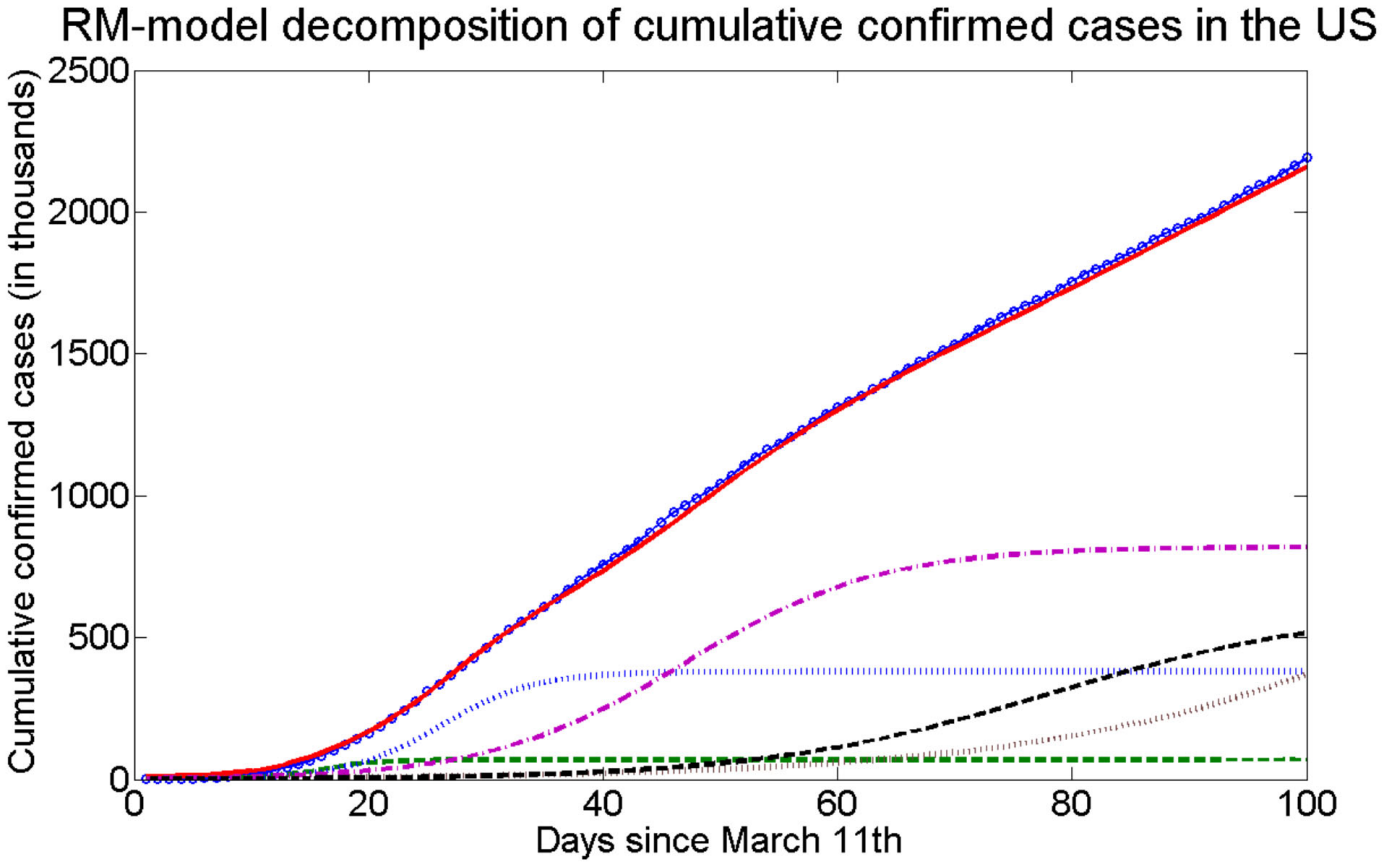
Cumulative confirmed cases in the US from March 11th to present time of June 18th (blue with circles) and total concatenated-RM model prediction (red), along with the predictions of the five RM components (green-dashed, blue-dotted, purple-dot-dashed, brown-dotted, and black-dashed).

Fig. 1 shows the cumulative number of confirmed cases in the US since March 11th 2020 along with the total model prediction and the predictions of the five RM components. The depicted RM-decomposition of the time-course of the cumulative number of confirmed cases offers useful insight into the time-course of the epidemic unfolding over five major IPs (defined as the source of statistically significant RMs) in the US between March 11th and the present time (June 18th). Consistent with the estimates shown in Table I, Fig. 1 indicates that the last RM model component is expected to make the largest contribution to the total number of confirmed cases, relative to the previous four RMs (see Discussion).

**Fig. 2.**
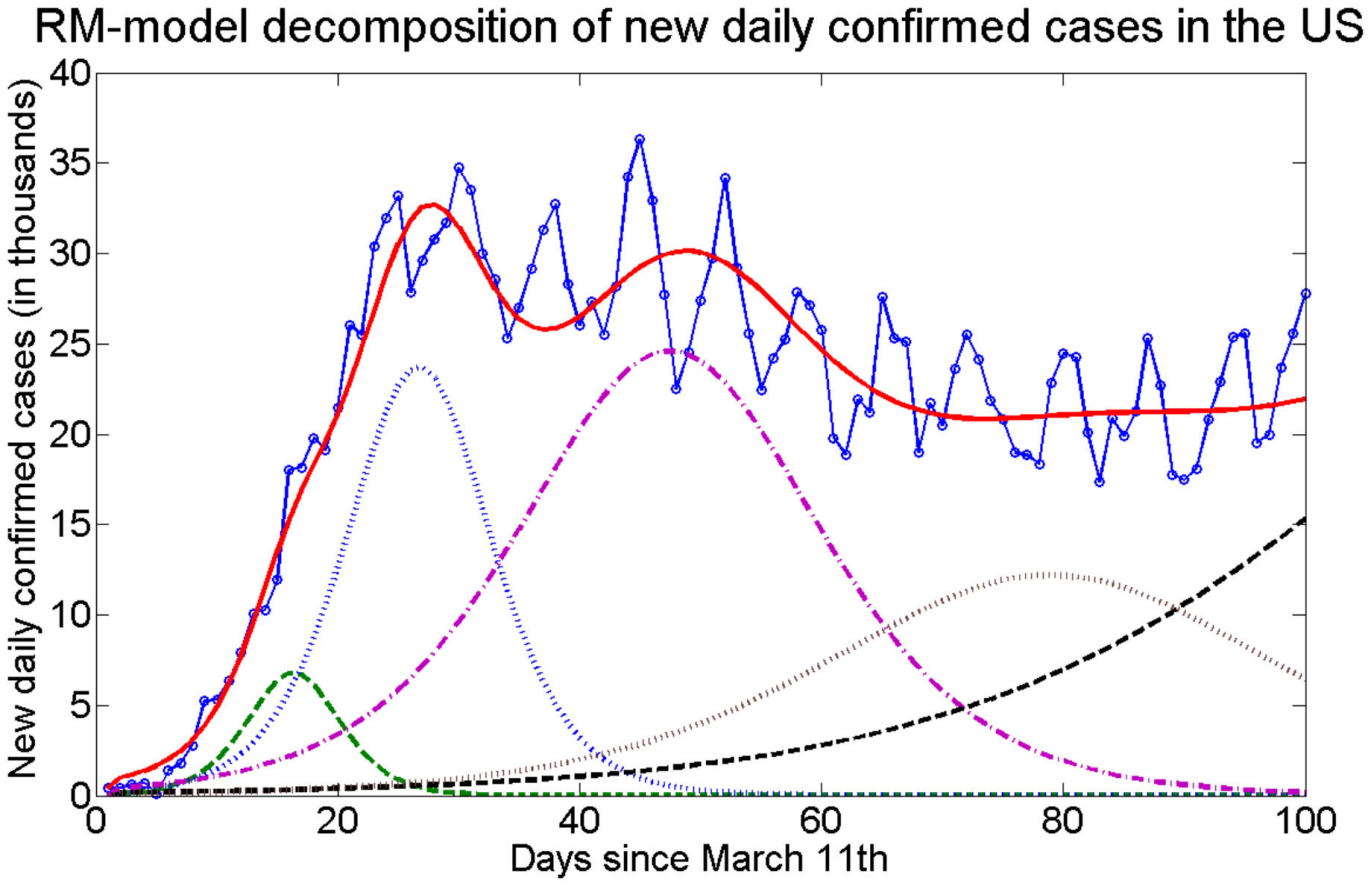
New daily confirmed cases in the US from March 11th to present time of June 18th (blue with circles) and the total concatenated-RM model prediction (red), along with the predictions of the five RM components (green-dashed, blue-dotted, purple-dot-dashed, brown-dotted, and black-dashed).

The analysis of the daily new confirmed cases offers an informative RM-decomposition that is shown in Fig. 2, along with the actual time-series data and the total model prediction. This result demonstrates the ability of the proposed approach *to model multi-modal patterns of dynamic changes in the infectious process due to merging of distinct infection pools – unlike the unimodal patterns of* the widely used SIR models. This also allows the timely detection of emerging distinct waves of infection (see Discussion).

The number of daily new confirmed cases due to each RM is given by the expression:

\begin{equation*}
\boldsymbol {\Delta X} \left(\boldsymbol {n} \right) = \boldsymbol {A}^{\boldsymbol {2}} \boldsymbol {K}\ \mathbf {exp} \left( - \boldsymbol {An} \right)/\{ \boldsymbol {B}{\left[ {\boldsymbol 1 + \boldsymbol{K}\ \mathbf {exp} \left( - \boldsymbol {An} \right)} \right]}^{\boldsymbol 2} \} \tag{9}
\end{equation*}

that exhibits a single peak at the PIR time-point ${\boldsymbol T_{PIR}}$ (see Equations (7) and (8)), which corresponds to the inflection point of the respective sigmoidal curve and is half-way to the level of the sigmoidal plateau (i.e. foretells the maximum value of cumulative cases to be reached by each RM).

**Fig. 3.**
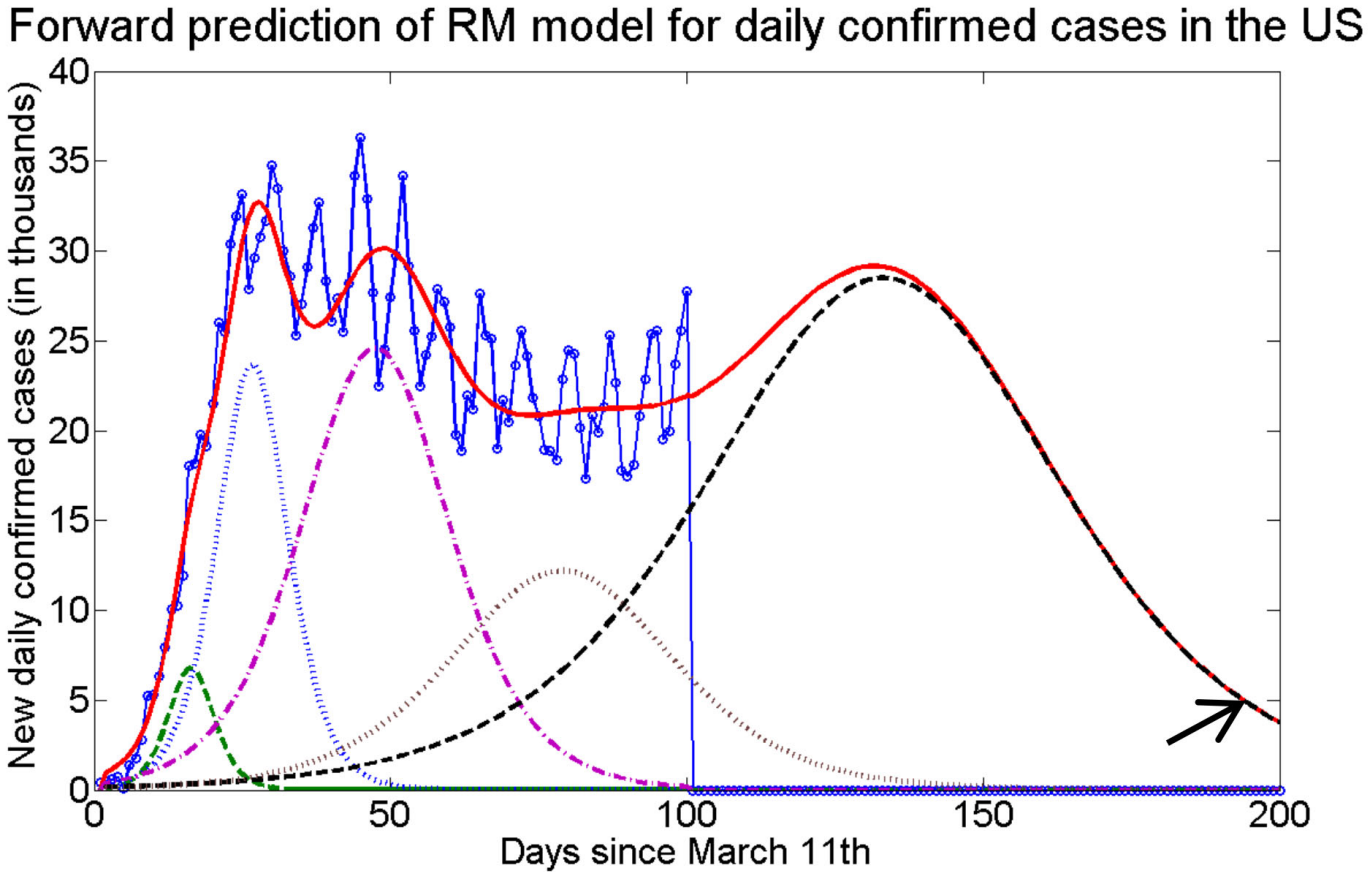
Forward prediction of the RM-based model for the new daily confirmed cases in the US over the next 100 days (to September 26th) made on June 18th (red line), along with the actual time-series data to date (blue with circles) and the predictions of the five RM components (green-dashed, blue-dotted, purple-dot-dashed, brown-dotted, and black-dashed).

It is evident in Fig. 2 that the first four RMs have passed their PIR time-points (see Table I). The last RM is expected to reach its PIR time-point on Day 132 (i.e. on July 20th). This RM-based model predicts that, unless a new IP is recruited in the near future, the Covid-19 infection in the US will dip below 5,000 new daily confirmed cases on Day 194 (i.e. on September 20th), as marked with an arrow in Fig. 3 that shows the simulated prediction of the five RM model components over the next 100 days (until September 26th). It is evident in Fig. 3 that the infection wave of the last RM is expected to be larger than the combined total of the other four RMs (see Discussion).

**Fig. 4.**
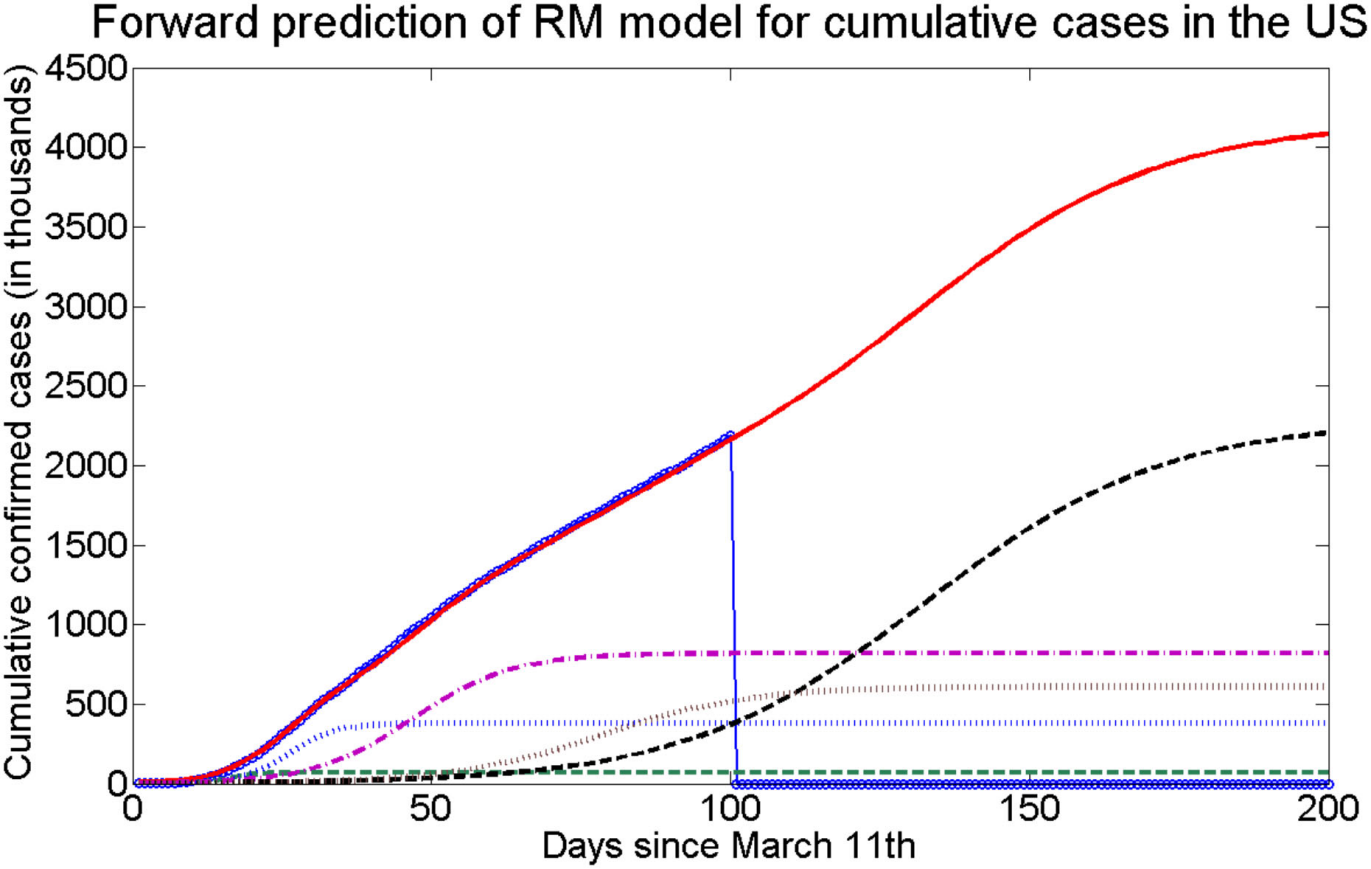
Forward prediction of the RM-based model for the cumulative confirmed cases in the US over the next 100 days (to September 26th) made on June 18th (red line), along with the actual data to date (blue with circles) and the predictions of the five RM components (green-dashed, blue-dotted, purple-dot-dashed, brown-dotted, and black-dashed).

The forward prediction of the RM-based model for the cumulative confirmed cases in the US over the next 100 days (provided that no new infection wave emerges) is shown in Fig. 4 and illustrates the dominant contribution of the last infection wave that has not yet reached its inflection point $({\boldsymbol T_{PIR}})$ that is expected in 32 days (i.e. on July 20th).

**Fig. 5.**
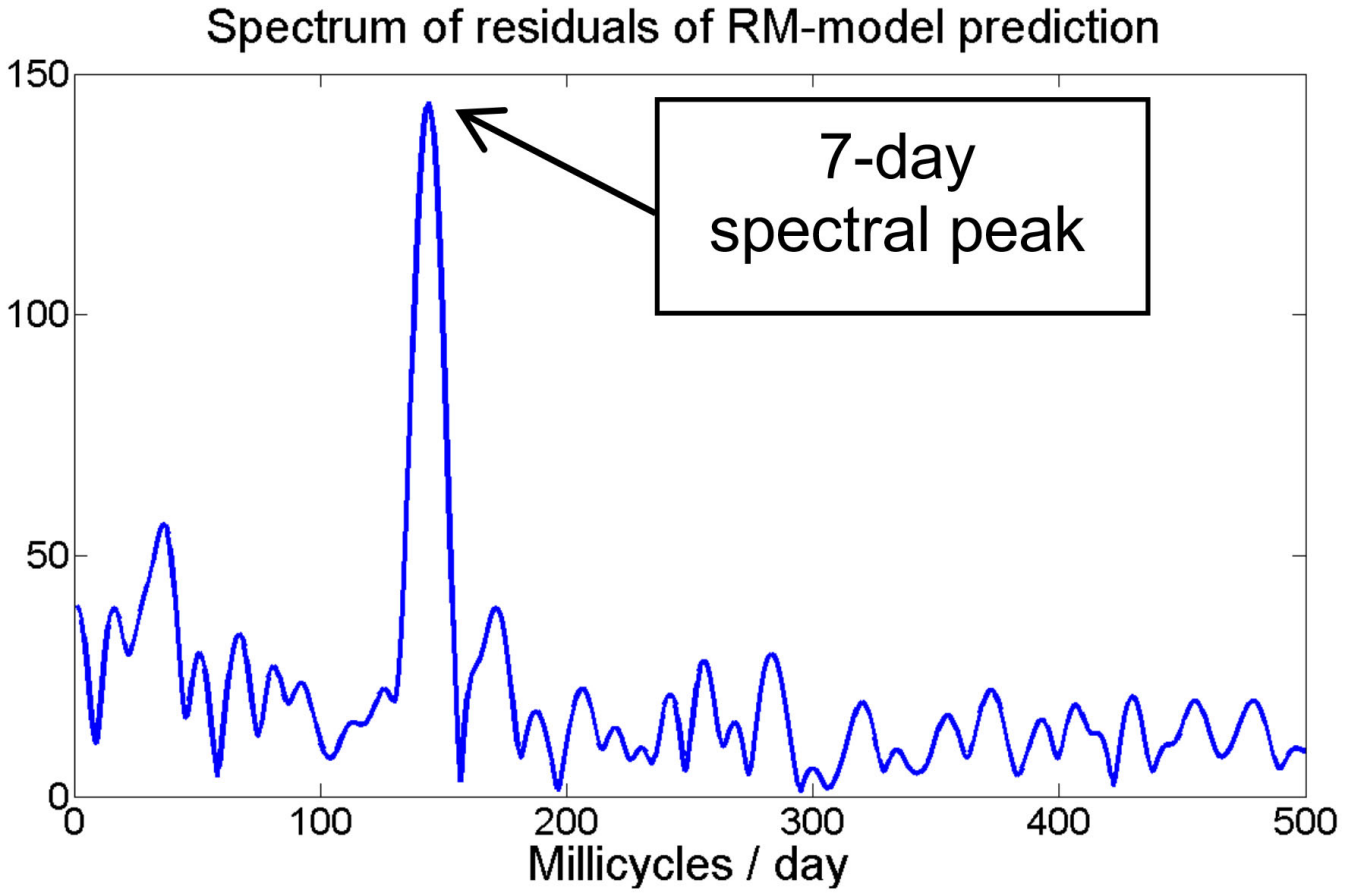
The frequency spectrum of the residuals of the RM model prediction for the new daily confirmed cases in the US that depicts a 7-day spectral peak at 143 millicycles/day.

A cyclical ripple is evident in the actual data of daily confirmed cases in Fig. 3 that is not accounted by the RM-based model. It is probably due to time-varying influences related to the weekly cycle of social life. The RM-based model is not expected to account for such time-varying influences, although the use of the fundamental Riccati Equation (1) can be extended in future work to time-varying coefficients ***A*** in order to account for these weekly variations. To examine the dominant frequencies of these variations, Fig. 5 shows the frequency spectrum of the residuals of the RM model prediction for the daily confirmed cases that clearly depicts a 7-day spectral peak (located at 0.143 cycles/day).

**Fig. 6.**
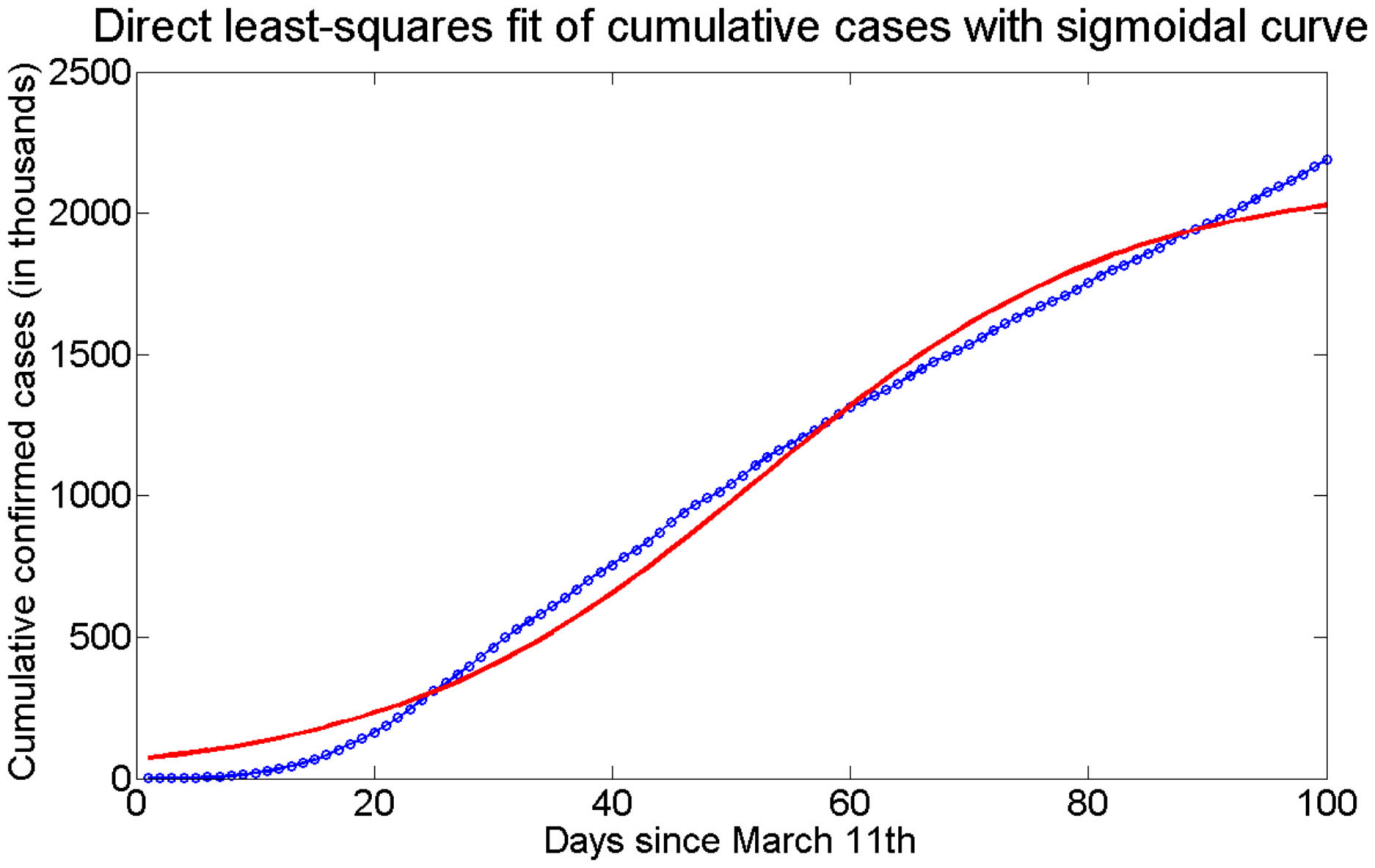
Direct least-squares fit (red line) of the cumulative cases of confirmed Covid-19 patients in the US from March 11th to June 18th (blue line with circles). The results are inferior to their counterparts from the proposed RM-based modeling methodology that are shown in Fig. 1.

Finally, since some take the view that simple curve-fitting of the cumulative cases data to a sigmoidal expression may be adequate, we examine whether a direct least-squares fitting of the sigmoidal expression of Equation (2) to the time-series data of cumulative confirmed cases may be able to yield a reasonable approximation of the time-course of the data. The result is shown in Fig. 6 and demonstrates the inferiority of simple curve-fitting, both in terms of approximation accuracy (by comparing with the RM-model approximation in Fig. 1) and in terms of misleading parameter estimates: low infectivity rate estimate of ${\boldsymbol A_{sig}}= 0.065$ and low prediction of maximum number of confirmed cases: 2,120,000.

**Fig. 7.**
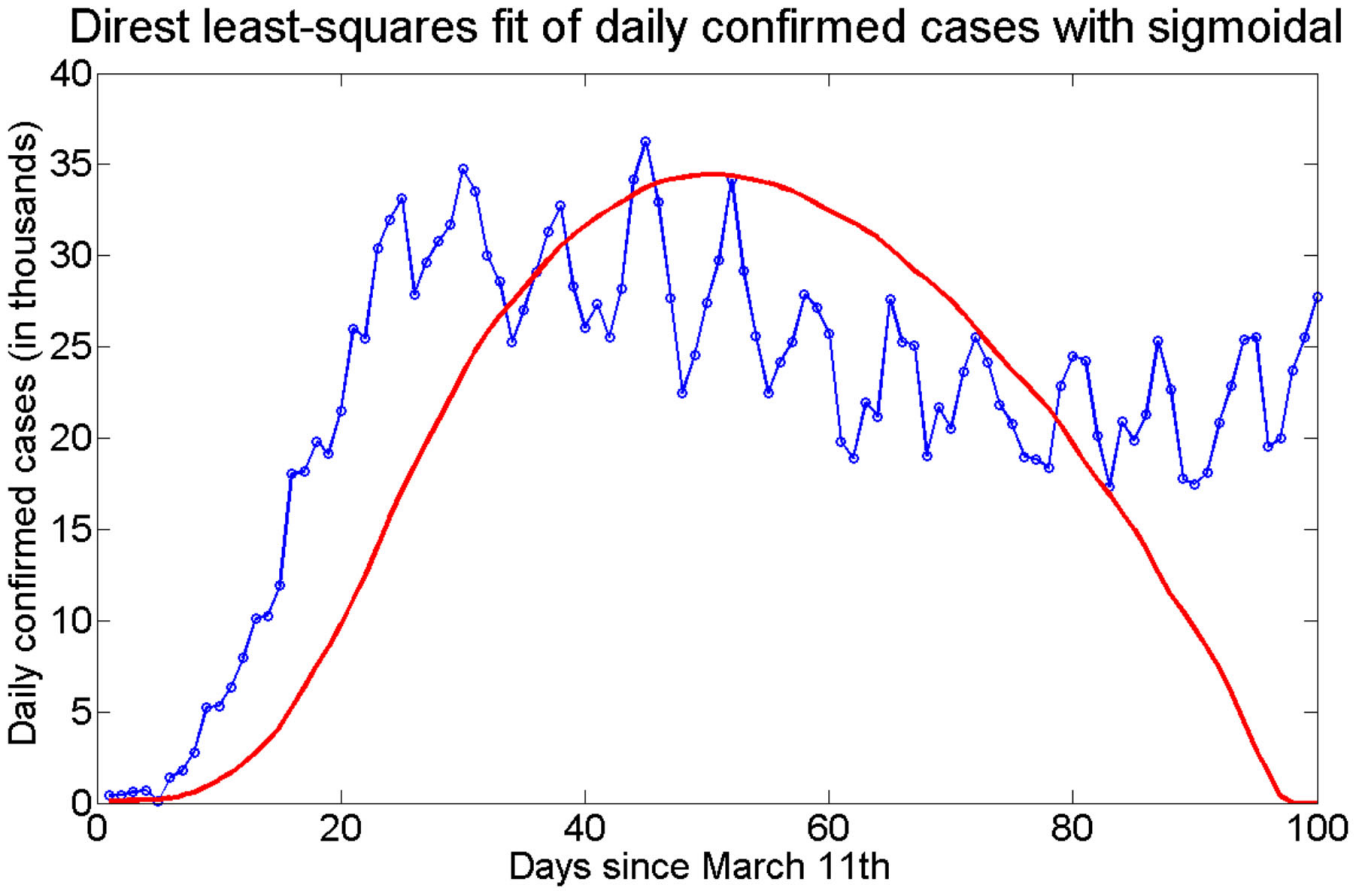
Direct least-squares fit (red line) of the daily cases of confirmed Covid-19 patients in the US from March 11th to June 18th (blue line with circles). The results are inferior to their counterparts from the proposed RM-based modeling methodology that are shown in Fig. 2.

For the data of daily confirmed cases, the direct least-squares approximation is shown in Fig. 7 and demonstrates the inferiority of curve-fitting in terms of approximation accuracy (by comparing with the RM-model approximation in Fig. 2) and the fundamental inability of direct sigmoidal fitting to approximate multi-modal phase-plots that can detect the emergence of new major infection waves.

## IV. Discussion & Conclusion

A novel adaptive methodology for predictive modeling of the time-course of daily and cumulative confirmed cases of Covid-19 has been presented and its application to the reported data for the US has been demonstrated. This methodology achieves the decomposition of the time-course of the Covid-19 data in terms of concatenated “Riccati Modules” (RM) and provides potentially useful predictions as well as valuable insights into the dynamic characteristics of the infectious process.

**TABLE II:** Units for Magnetic Properties

Symbol	Quantity	Conversion from Gaussian and CGS EMU to SI^a^
Φ	magnetic flux	1 Mx → 10^−8^ Wb = 10^−8^ V·s
*B*	magnetic flux density, magnetic induction	1 G → 10^−4^ T = 10^−4^ Wb/m^2^
*H*	magnetic field strength	1 Oe → 10^3^/(4π) A/m
*m*	magnetic moment	1 erg/G = 1 emu → 10^−3^ A·m^2^ = 10^−3^ J/T
*M*	magnetization	1 erg/(G·cm^3^) = 1 emu/cm^3^ → 10^3^ A/m
4π*M*	magnetization	1 G → 10^3^/(4π) A/m
σ	specific magnetization	1 erg/(G·g) = 1 emu/g → 1 A·m^2^/kg
*j*	magnetic dipole moment	1 erg/G = 1 emu → 4π × 10^−10^ Wb·m
*J*	magnetic polarization	1 erg/(G·cm^3^) = 1 emu/cm^3^ → 4π × 10^−4^ T
χ*,* κ	susceptibility	1 → 4π
χ_ρ_	mass susceptibility	1 cm^3^/g → 4π × 10^−3^ m^3^/kg
μ	permeability	1 → 4π × 10^−7^ H/m = 4π × 10^−7^ Wb/(A·m)
μ_r_	relative permeability	μ → μ_r_
*w, W*	energy density	1 erg/cm^3^ → 10^−1^ J/m^3^
*N, D*	demagnetizing factor	1 → 1/(4π)

Vertical lines are optional in tables. Statements that serve as captions for the entire table do not need footnote letters.

^a^Gaussian units are the same as cg emu for magnetostatics; Mx = maxwell, G = gauss, Oe = oersted; Wb = weber, V = volt, s = second, T = tesla, m = meter, A = ampere, J = joule, kg = kilogram, H = henry.

Specifically, the advocated approach detects the presence of multiple overlapping “infection waves” that correspond to major “infection pools” (IP) described by distinct and concatenated RMs that are defined by the fundamental Riccati Equation (1) – each with distinct parameters ***A*** and ***B*** that quantify the critical dynamic aspects of the infectious time-course in the respective IP. The parameter ***A*** is the “infectivity rate constant” that determines the initial exponential-like growth of the infection and depends on the degree of contagiousness and the level of contagious interactions in a given IP. In this sense, it is akin to the “reproduction rate” of the conventional SIR models. The parameter ***B*** depends on the size of the susceptible and exposed population in each IP and also quantifies the degree to which the gradually acquired “herd immunity” and mitigating factors/measures constrain the initial rapid growth of the infection and eventually achieve its control according to the sigmoidal time-course defined by Equation (2) reaching at its plateau the maximum number of infections: ${\boldsymbol X_{\max}={ (\boldsymbol {A/B})}}$.

To achieve this RM-decomposition of the time-series data, the proposed approach employs regression analysis in phase-space and statistical Hypothesis testing using an F-statistic (see Methods) to detect the emergence of new infection waves at a specified level of statistical significance. Running (adaptive) estimates of the RM parameters are obtained at each time-point. They were found to be rather stable away from the points where new RMs are introduced into the model.

Analysis of Covid-19 daily data in the US from March 11th to June 18th (when this manuscript was completed) yielded five RMs that are concatenated as shown in Figs. 1 and 2. They are deemed to represent the distinct dynamics of five infection waves in major IPs that have the characteristics defined by their respective parameters given in Table I. The small initial RM-1 (possibly corresponding to the initial infection in the Seattle area) is followed by the larger RM-2 and RM-3 (possibly corresponding to the rapid urban surge in New York City and subsequently in other US urban centers and the Northeast, respectively). The broader epidemic spread across smaller towns and rural areas in the US, under local mitigation measures, may correspond to RM-4 (slower growth and moderate size). The emergence of the last and largest infection wave (described by RM-5) was detected by the proposed algorithm on Day 60 (May 9th) and appears to coincide with the relaxation of some mitigation measures across the US. The total number of infections anticipated by the model is 4,160,000 (about double the current cumulative number), provided that there will be no new RM added to the model because of Covid-19 spreading into a new IP or caused by significant change in the current mitigation measures. Under the same key assumptions, the current model predicts that the number of new confirmed cases in the US will drop below 5,000 by September 20th (see Fig. 3).

The results shown in Table I and Fig. 2 indicate an early rapid reduction of the parameter ***A*** in successive RMs, which plays a key role in determining the critical “stressor” of the healthcare system, the Peak Infection Rate: ***PIR = A^2^/(4B)***, provided that the parameter ***B*** is not drastically reduced. The last RM anticipates its ***PIR*** to occur in 32 days (July 20th) without exceeding the previous peaks of RM-1 and RM-2. It is worth noting that the time between detection of a new infection wave and its ***PIR*** increases with decreasing ***A***.

Analysis of the daily confirmed cases shows the individual contributions of the five RM components (see Fig. 2) and demonstrates the versatility of the proposed approach to detect in a statistically rigorous manner new emerging waves of infection and be applicable to cases where the pattern of daily changes is *not unimodal.* This constitutes an important advantage of the proposed approach over the widely used SIR models and other unimodal approaches. Another difference of the proposed approach from the popular SIR model is that it does not take into account the number of recovered cases and does not require full immunity of the latter. To further explore this comparison, the three equations of the classic SIR model can be combined in a single nonlinear differential equation that takes the second-order form:

\begin{equation*}
\boldsymbol {d}^{\boldsymbol 2} \boldsymbol {Q} / \boldsymbol {dt}^{\boldsymbol 2} + \boldsymbol {k}\ \boldsymbol {dQ} / \boldsymbol {dt} = \boldsymbol {s}_{\boldsymbol 0} \boldsymbol {b}\ \mathbf {exp} \left[ - \boldsymbol {b} \ \boldsymbol {Q} \left(\boldsymbol{t} \right) \right]\tag{10}
\end{equation*}

where ***Q(t)*** is the integral from 0 to ***t*** of the infected fraction of the population, ***k*** is the recovery rate, ***b*** is the infection rate and ***s*_0_**is the initial size of the susceptible population. Equation (10) indicates that the estimation of the unknown parameter ***b*** must rely on iterative methods (which are far less robust and reliable than regression utilized by the proposed approach) and that this differential equation has only one stable equilibrium point when ***Q(t)*** tends to infinity (a less flexible notion than the multiple finite stable equilibrium points of the concatenated Riccati Equations that are achieved by each RM when each reaches its individual plateau for the respective ${\boldsymbol X_{\max}= {\boldsymbol{A/ B}}}$). These comparisons must be explored further in the future.

Regarding the cyclical variations that are evident in the time-series data of daily confirmed cases, but not accounted by the RM-based model (see Fig. 3), it is noted that the fundamental Riccati Equation (1) can be extended in future work to time-varying coefficients that may account for the observed 7-day cycle revealed in the spectrum of the residuals of the model prediction (see Fig. 5). The 7-day cycle peaks at the end of each week and may be due to increased social interactions during the previous weekend (noting the average Covid incubation period of 5 days).

It must be emphasized that the RM-based predictive modeling is distinct from simple curve-fitting methods. This was demonstrated above by contrasting with the results of direct sigmoidal least-squares fitting (see Figs. 6 and 7) and showing that the latter may lead to serious mis-estimation of the key parameters of the infectious process (e.g. much smaller infectivity rate estimate and smaller predicted maximum number of confirmed cases) – in addition to misconceptions regarding the dynamic structure of the process (i.e. unimodal versus multi-modal phase-space representation).

An interesting question arises with respect to the effect of changing testing rates upon the obtained parameter estimates. If the “true” incidence is ***Y(t)***, then the “apparent” incidence due to a time-varying “testing rate function” ***f(t)*** is: ***X(t) = f(t)Y(t)***. It can be shown that the “true” parameters A^*^ and B^*^ (corresponding to the unknown ***Y(t)*** values) are related to the “apparent” parameter estimates ***A*** and ***B*** (obtained from the available ***X(t)*** data) according to the expressions: A = A^*^ + f’(t)/f(t), and B = B^*^/ f(t), where ***f’(t) = df(t)/dt***. Since ***f(t)*** ought to be positive and ≤1 for all times, then ***B*** is always an overestimation of B^*^, and ***A*** overestimates A^*^ only when the testing rate is increasing (***f’(t) >0***). For a constant testing rate, A = A^*^. For the estimated maximum number of cases, we have the relation: ${\boldsymbol X_{\boldsymbol \max}= {\boldsymbol Y}_{\boldsymbol \max}\,\,[ \boldsymbol {f(t)+ f\prime (t)\,/ \,A^{\ast}}] }$.

This work (like others on Covid-19 predictive modeling) is published under unique and unprecedented circumstances of an ongoing pandemic, which render its validation open to the future data that are publicly reported. The predictions made in this paper will *hold only if no new wave of infections occurs*.

The proposed approach can be applied in the near future to additional Covid-19 data from other countries or from various regions of the US in order to compare the obtained RM-decompositions (revealing the dynamic structure of infection waves in these infectious processes) and the associated parameter estimates ***A*** and ***B*** of each RM. The distinct RM-decompositions for various countries/regions and the respective parameter estimates may reveal valuable correlations with the mitigation policies followed in each case to examine their effectiveness within each specific socio-cultural context in order to guide future decision making by examining how much the respective policies or socio-cultural conditions influence the estimated parameters ***A*** and ***B*** – and consequently ${\boldsymbol X_{\max}= {\boldsymbol{A/B}}}$ or ***PIR = A^2^/(***4***B)***.
